# Fracture Resistance of Fiber-Reinforced vs. Conventional Resin Composite Restorations in Structurally Compromised Molars: An In Vitro Study

**DOI:** 10.1155/ijod/5169253

**Published:** 2025-02-25

**Authors:** Hamideh Sadat Mohammadipour, Mostafa Farajzadeh, Hediyeh Toutouni, Arya Gazerani, Salehe Sekandari

**Affiliations:** ^1^Department of Restorative Dentistry, School of Dentistry, Mashhad University of Medical Sciences, Mashhad, Iran; ^2^Department of Restorative Dentistry, School of Dentistry, Isfahan University of Medical Sciences, Isfahan, Iran; ^3^Department of Community Oral Health, School of Dentistry, Mashhad University of Medical Sciences, Mashhad, Iran; ^4^Dental Materials Research Center, Department of Restorative Dentistry, School of Dentistry, Mashhad University of Medical Sciences, Mashhad, Iran

**Keywords:** cusp capping, cusp coverage, cusp reduction, fracture mode, fracture resistance, fracture strength, resin composite, short fiber-reinforced resin composite

## Abstract

**Objective:** The aim of this study was to evaluate reinforcing effect of different fiber-reinforced resin composites for restoring structurally compromised molars compared to conventional resin composite.

**Methods and Materials:** Sixty healthy human third molars were randomly divided into six groups (*n* = 10). In G1 to G4, wide mesio–occluso–distal (MOD) cavities with an occlusal depth of 4 mm and proximal boxes with a width of 2/3 of buccolingual distance were prepared. In G5, after preparation of MOD cavities, 2 mm cusp reduction was made. The samples of G6 were remained intact (control). In G1 and G5, the conventional resin composite (G-ænial Posterior), and in G2, short fiber-reinforced resin composite (SFRC: EverX Posterior) were used. In G3 and G4, the Ribbond fibers were applied on base of cavity in buccolingual and cross-sectional direction, respectively, and followed by conventional resin composite restoration. After 24 h maintaining in distilled water at room temperature, fracture resistance of restored teeth was tested with a crosshead speed of 0.5 mm/s and fracture patterns were also evaluated by stereomicroscope (favorable: above cementoenamel junction (CEJ) and unfavorable: below CEJ). The data was analyzed using Shapiro–Wilk, one-way ANOVA, and post hoc Tukey's HSD tests.

**Results:** The highest fracture strength was obtained in G2 (4051.4 ± 1293.9 N), which was significantly greater than G3 (2886.6 ± 720.4 N; *p* = 0.005) and G5 (2949.3 ± 307.5 N; *p*  = 0.010). No statistically significant difference was observed between other study groups. The greatest percentage of favorable fracture was recorded in G6 (70%) and G2 (60%).

**Conclusion:** The reconstruction of severely weakened molar teeth with SFRC or incorporating of polyethylene fibers in cross-sectional direction on base of resin composite restoration improved fracture strength and favorably affected fracture modes in comparison to conventional posterior resin composite with or without cusp coverage.

## 1. Introduction

The amount of remaining tooth structure, especially proximal walls, in endodontically treated posterior teeth is an important factor in the strength, durability, and long-term survival rate of a restoration [1]. However, in extensive cavities, with an isthmus width of more than two-third of the intercuspal distance, cusp coverage is more important than preserving the weakened structure [2].

An ideal restorative material should not only restore the defective structure and create an adequate seal between the restoration and the tooth but also provide the strength of the tooth. Theoretically, resin bonding with dental tissue and its function as an internal splint can restore the lost fracture strength of the tooth [3]. However, the strengthening effect of intracoronal direct resin composite restoration when used alone for large cavities is challenging [4]. It should be considered that in extensive posterior cavities, the use of conventional resin composite materials due to their insufficient fracture strength and polymerization shrinkage is associated with possibility of composite or tooth fracture, secondary caries, and marginal defects [5].

Biomimetic approach in restorative dentistry is to study the structure and function of tooth tissue as a model for the design and production of materials or techniques that can be used to restore tooth with the least invasive actions, while mimicking its mechanical characteristics [4]. Using different types of fibers with different sizes and orientations under resin composite, in order to achieve greater hardness and fracture strength, is an idea that has been considered for a long time [6]. Fiber reinforced composite (FRC) materials, as their name suggests, are a combination of polymer matrix and reinforcing fibers. The presence of fibers in the polymer matrix creates paths that lead and withstand the stress caused by occlusal forces [7]. These reinforcing fibers can be placed in different orientation patterns [6]. Various studies have shown the FRCs can increase the fracture strength [7–13] and the resistance to microleakage by reducing polymerization contraction [3, 7, 14, 15].

During the last few decades, efforts have been made to achieve better mechanical properties for resin composite materials. By incorporation of short fibers into the resin materials structure and introducing of short fiber-reinforced resin composites (SFRCs) in dental market, it can be possible to strengthen the composite restorations in the posterior prestressed areas [6, 16–18]. It has been shown that they can restore the strength of the tooth's internal structure and prevent the tooth fractures by mimicking the stress absorption characteristics of dentin which provides more reliable and conservative composite restorations [7, 8, 19]. Other features of SFRCs include higher flexural strength and bond durability, in addition to reducing microleakage and improving marginal integrity [4, 20]. Also, these materials are easy to use and are considered effective for restoring dentin in terms of time, although their strengthening may not be as strong as long fibers [17].

During preparation of the extensive cavities with thin and weak external walls and marginal ridges (thickness less than 2 mm) [21], it is necessary to shorten the thin walls and then replace these areas with a restorative material in cusp coverage process. Several studies suggest that cusp coverage along with resin composite materials minimizes tooth fracture and increases the durability of restorations [22–25]. Although, this procedure increases the resistance form, it is adversely affected the retention form which has a great importance in endodontically treated teeth. So, the majority of previous studies focused on the endodontically teeth and the effect of SFRCs and there is not enough information about effect the polyethylene fibers on strength of nonendodontically teeth with extensive cavity preparation. Therefore, this study was designed to evaluate the reinforcing effect of polyethylene fibers and SFRCs material in large mesio–occluso–distal (MOD) class two(Cl II) restorations and compared them with conventional cusp coverage and restoration with conventional posterior resin composite material. The current research evaluated the null hypothesis that there was no significant difference between utilizing conventional posterior resin composite with and without polyethylene fibers and SFRC in extensive class two cavities in molar teeth with and without cusp coverage.

## 2. Methods & Materials

### 2.1. Study Design

This in vitro study was conducted at the Department of Restorative Dentistry (Mashhad University of Medical Sciences, Mashhad, Iran). The local ethical committee of Mashhad University of Medical Sciences, Iran, independently reviewed and approved this in vitro study with the protocol number of IR.MUMS.DENTISTRY.REC.1401.093.

Because a study similar to the current study has not been conducted, the study was first started as a pilot study with 10 samples, after analyzing the initial results, sample size was calculated based on the following formula for multiple mean comparison using one-way ANOVA:

  $$\begin{array}{c} n=\frac{\lambda_{g,\alpha ,1-\beta }}{\Delta }, \end{array}$$

in which *g* = number of groups, *α* = 0.05, 1 − *β* = 0.80, resulted in *λ*_*g*,*α*,1 − *β*_ = 12.83.

*Δ* was calculated using: $\Delta =\frac{1}{\sigma^{2}}\sum_{i=1}^{k}{\left(\mu_{i}|-|\mu^{-}\right)}^{2}$ = 1.5633

The final sample size was 8.2 and due to the fact that it was less than the initial sample number, the study was terminated.

### 2.2. Sample Preparation

Sixty sound and intact third molar teeth, which were extracted for periodontal and orthodontic reasons were collected. The tissue debris was removed by a scaler (LM-Dental, Parainen, Finland), the teeth were cleaned under running tap water, and then examined by a stereomicroscope (Dino lite Pro, Anmo Electronic, New Taipei City, Taiwan) under magnification ×10 to exclude those with caries, erosion, enamel cracks, deep grooves, or fractures. The teeth were kept in 0.1% thymol solution for 1 week and placed in 0.9% saline solution at room temperature until the initiation of the experiment. All samples were tested within 2 months after extraction. The samples were selected in the range of the similar buccolingual, mesiodistal, and occlusocervical dimensions with a standard deviation (SD) of ±10% from the mean (maxillary molars: buccolingual dimension 10 mm and mesiodistal dimension 9 mm; mandibular molars: buccolingual dimension 9 mm and mesiodistal dimension 10 mm). A vernier caliper was used to measure the molars, and one person performed the measurements.

### 2.3. The Cavity Preparation

Each tooth was placed vertically in the center of a custom-made cylindrical mold in self-cured methacrylate resin (Acropars, Marlic Medical Industries Co., Tehran, Iran) to a distance of 1 mm from the cementoenamel junction (CEJ). The teeth were randomly divided into six groups (*n* = 10).

In groups 1–4 (G1–G4), the standard MOD Cl II cavities with an occlusal depth of 4 mm from the central fissure and 1 mm above the CEJ at the cervical aspect of the proximal boxes were prepared. In 90% of the samples, the proximal depth was 6 mm, and in other specimens were 5 or 7 mm. The width of proximal boxes was two-thirds of the buccolingual distance in the mesial and distal surfaces in which the thickness of 2 mm remained in the buccal and lingual walls. All cavity walls were prepared parallel to each other with a 90° cavosurface angle. No beveled edges were apparent on the cavosurface angles of the preparations. The cavity preparation was done by a diamond fissure bur (FGG111010 DIAMIR s.r.l, Via Prato, Resia, Italy) in high-speed handpiece with water and air spray. The cavities were prepared and checked by one operator who ensured the same size of the cavities by using a UNC-15 probe (Hu-Friedy Mfg. Co., Chicago, USA) and standard burs.

**Figure 1:**
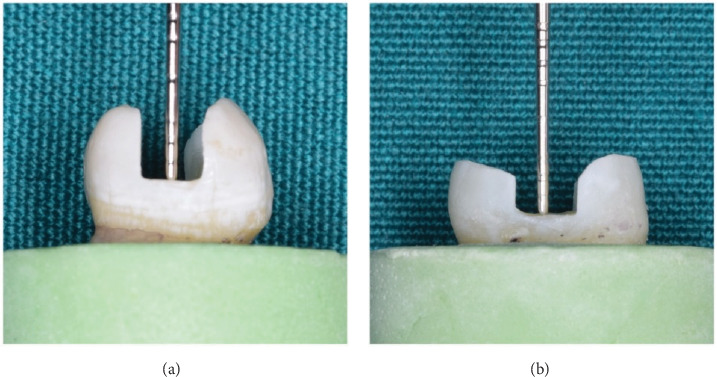
The proximal view of the cavity preparation: (a) The cavity without cusp reduction (in groups 1–4) and (b) the cavity with cusp reduction (group 5).

In group 5 (G5), after preparation of described MOD Cl II cavities, the cusp reduction was made by 2 mm reduction of the buccal and lingual walls. Each bur was used for five cavity preparations and then replaced by a new one. After cavity preparation, all cavities were washed and air-dried and were ready for restorations (Figure 1).

The samples of control group (G6) were remained intact.

### 2.4. The Bonding Procedure

The enamel walls were etched for 30 s and the dentin walls for 15 s by 37% phosphoric acid gel (Condac, FGM, Joinvile, SC, Brazil). Subsequently, they were rinsed with a large amount of water and then air-dried. It was ensured that the enamel had a frosty appearance and the dentin remained slightly moist and not dehydrated. Then, according to the manufacturer's instructions, two consecutive layers of adhesive (Adper Single Bond 2, 3M ESPE, St. Paul, MN, USA) were applied. After 10 s of evaporation of the solvent by air pressure, the adhesive was cured for 10 s with an LED-B curing device (Guilin Woodpecker Medical Instrument Co., Guangxi, China) with a power of at least 650 mW/cm^2^. The intensity of the light cure device was checked at first and after every 10 exposures by a digital radiometer device, Woodpecker Curing Light Power Meter (LM-1, Guilin Woodpecker Medical Instrument Co., Guangxi, China).

All MOD cavities were first converted into a Class I cavity by using a metal matrix band and Tofflemire Matrix Retainer (Hahnenkratt, Konigsbach-Stein, Germany) and placing two layers of posterior G-ænial resin composite (CG, Tokyo, Japan) on the mesial and distal side of the cavity and curing of each layer for 20 s. The remaining parts of the cavities were restored as follows:

Group 1 (G1): The entire cavity was filled with two horizontal layers of G-ænial Posterior resin composite, and each layer was light-cured for 20 s.

Group 2 (G2): EverX Posterior resin composite (CG Corp., Tokyo, Japan) was placed inside the cavity using a composite injection gun (Cavifill Injector, Ivoclar Vivadent, Schaan, Liechtenstein) through the bulk-fill technique to fill 3 mm of the cavity height. After 20 s of light polymerization, the occlusal surface (1 mm remaining part of the cavity) was filled with a 1 mm thick G-ænial Posterior resin composite layer and polymerized.

Group 3 (G3): In this group, the proper length of polyethylene fibers (Ribbond Ultra-THM, Ribbond Inc., Seattle WA, USA) that was 2 mm longer than the buccolingual length of the cavity were cut by special scissors (Ribbond Inc., Seattle WA, USA) and were then put on the pulpal floor of the cavity in the buccolingual direction to extend 1 mm on each buccal and lingual wall. Then, the fibers were fixed by the flowable resin composite (Charisma Flow, Kulzer, Germany) and the resin composite was polymerized. Finally, the rest of the cavity was filled with G-ænial Posterior resin composite, similar to the first group.

Group 4 (G4): The restoration method of this group was similar to the third group, with the difference that the ribbons were used in the form of cross-links in the mesiodistal and buccolingual directions in the pulpal floor of the cavity. For this purpose, the first ribbon was placed in the buccolingual direction, similar to the third group, and after curing, the second fiber was placed in the mesiodistal direction equal to the internal distance between the proximal composite walls. The restoration of the rest of the cavity was completed similarly to the first group with G-ænial Posterior resin composite.

Group 5 (G5): The restoration method in this group was similar to the first group, except that the reducted cusps were also reconstructed.

In all groups, immediately after the restoration, the finishing and polishing were done using fine and ultrafine diamond finishing burs (SS White Burs, Inc., Lakewood, NJ, USA) and the composite polishing kit (Compo Master, Shofu INC., Japan), respectively.

### 2.5. Fracture Strength Measurement and Fracture Type Assessment

**Figure 2:**
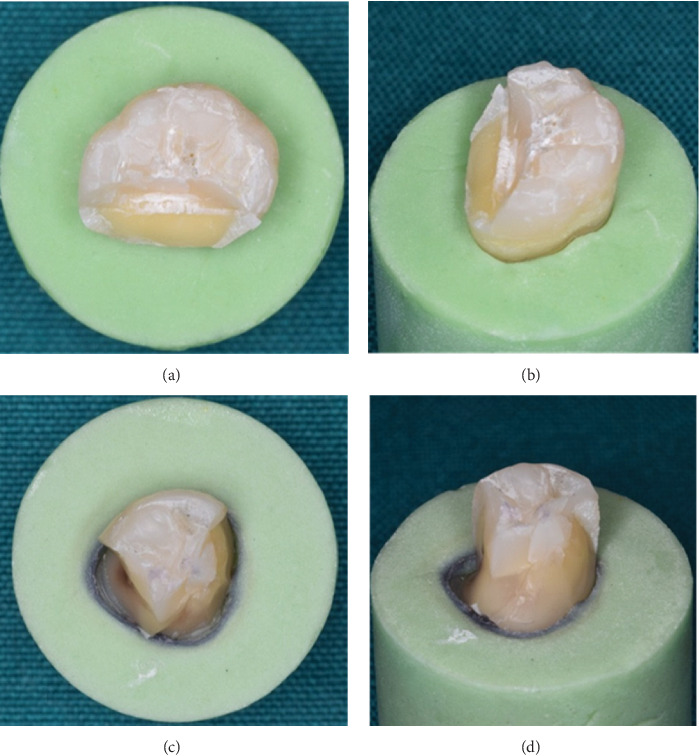
Examples of failed specimens. (a, b) Favorable/restorable fractures, where a mediate portion of the coronal part is fractured; but the fracture is still above the cementoenamel junction (CEJ). (c, d) When the fracture is below the CEJ and involves the root, making the fracture completely unfavorable/nonrestorable.

In all groups, to ensure the completion of polymerization of resin materials and the stability of the formed polymer network, the samples were maintained for 24 h in distilled water at room temperature. Then, a vertical compressive force through a cylindrical steel bar with 6 mm diameter and 10 mm long which was parallel to the longitudinal axis of the tooth and positioned at the center of the occlusal surface of the tooth crown between the buccal and lingual cusps [26] was applied using a Universal Testing Machine (Santam, model STM-20, Tehran, Iran) with a crosshead speed of 0.5 mm/min until the failure occurred. The amount of force at the time of failure which resulted in a peak formation on the extension curve was recorded as a fracture threshold for each sample in Newtons (N). The samples were classified according to the fracture area evaluated by stereomicroscope (Olympus BH2; Olympus Corp, Tokyo, Japan); fractures above the CEJ were considered favorable or restorable fractures, and the fractures below the CEJ were considered unfavorable or nonrestorable ones which are likely to be extracted [27, 28] (Figure 2).

### 2.6. Statistical Analysis

In this study, data were analyzed using the SPSS statistical software (version 24.0, IBM, Chicago, IL, USA). The Shapiro–Wilk test was used to assess the normality of data distribution. One-way ANOVA was also used for comparing the fracture strength of study groups. When the differences between the study groups were statistically significant, the Tukey's HSD test was used to show the differences between them. All the statistical analyses were performed with the statistical significance level of 5%.

## 3. Results

The results obtained from the Shapiro–Wilk analysis revealed the normal distribution of mean values (*p*  > 0.05). The descriptive data from the study groups including mean and SD values of fracture strength were presented in Table 1. Based on one-way ANOVA analysis, there was a significant difference between the study groups (*p* = 0.003). Tukey's HSD post hoc test revealed the mean fracture strength of G2 was significantly higher than G3 (*p* = 0.005) and G5 groups (*p* = 0.010). The comparison between the other study groups revealed no significant difference in fracture strength values.

The percentages of favorable and unfavorable fracture patterns were presented in Table 2. Regarding the fracture analysis, the highest percentage of favorable/restorable fractures in G6 and then in G2 and the greatest percentage of unfavorable/nonrestorable fractures in G5 were observed. The study groups in which the restorations were reinforced by Ribbond (G3 and G4) showed the same favorable and unfavorable fracture percentages.

## 4. Discussion

Nowadays, with the advances achieved in adhesive techniques and the emergence of high-strength resin composites, molar teeth with extensive destruction are easily reconstructed by resin materials [12]. Since, the results of this study showed there was a significant difference between the mean values of fracture strength between the study groups, the null hypothesis was rejected. Based on the obtained findings, SFRC (EverX Posterior) showed the highest fracture strength. Therefore, it can be confirmed that by conservative preparation (without cusp reduction) and restoration with EverX Posterior and the biomimetic approach to restore the role of dentin, it is possible to achieve fracture strength comparable to sound teeth or even more.

Cusp reduction leads the restoration margins to buccal and palatal surfaces, protecting the adhesive interface from marginal discrepancies [29], so resin composite restorations with cusp coverage can recover the strength of the weak teeth even to the values similar to the sound teeth, based on the previous studies [23, 30]. However, this approach could not improve the fracture strength of deep MOD cavities in this study and more unfavorable fractures happened in G5. One of the reasons for the difference in the results of our study can be the fact that the teeth we studied were vital teeth with much less cavity depth than the mentioned studies. Another justification for the results of current study can be the effect of this issue that the teeth in G5 had a cusp reduction, which represents a huge drawback for these teeth even with the cusp reconstruction, resulting in lower fracture resistance and more unfavorable fractures.

In this study, teeth restored with SFRC (G2) exhibited higher fracture strength than those restored with Ribbonds (G3 and G4). This finding aligns with earlier researches by Mangoush et al. [3] and Sah et al. [31], which indicated that SFRCs can enhance fracture strength to a level comparable to or even exceeding that of long fibers like Ribbond. However, it is important to note that the fracture resistance of restorations using cross-linked fibers was similar to that of SFRC in this study. The increased fracture strength observed in SFRC can be attributed to the crack-stopping properties of short E-glass fibers that are randomly oriented within the material, along with bonding effect of thin walls by these composites. The comparable fracture strength of G2 and G4 groups in this study in which the long Ribbond fibers placed in cross-sectional position inside the resin composite restorations, confirmed the role of fibers in improving the strength of weak teeth. Also, this stop of crack propagation towards the restoration center is consistent with the high percentage of favorable fracture patterns in G2.

In the present study, there was no significant difference between the fracture strength of restorations reinforced with Ribbond polyethylene fibers (G3 and G4) and the conventional restorations with or without cusp coverage (G1 and G5). The results suggest the possibility of decreasing cuspal fracture caused by cuspal deflection even without cusp coverage due to the splinting effect of bonding and composite restoration. This finding is similar to the outcome of a previous study which has been done by Cobankara et al. [32], in which there was no significant difference between reinforcement of Ribbond and conventional resin composite restoration in MOD cavities of endodontically treated teeth. In contrast, previous studies on endodontically treated teeth [9, 11, 33, 34] showed the use of Ribbond fibers to strengthen composite restoration causes a significant increase in fracture strength. The difference between their results with the present study may be related to the difference in the type of teeth (endodontically and nonendodontically teeth), the presence of access cavities, and the type of composites used in the above studies such as conventional nanohybrid resin composite and bulk-fill resin composite.

Among the study groups, the control teeth (G6) exhibited the highest percentage of favorable fractures, followed by the EverX Posterior group (G2) and then the Ribbond-reinforced groups (G3 and G4) and no significant difference was observed regarding the orientation of the fibers. The outcomes of our investigation corroborate the findings of numerous prior studies, which have indicated that restorations incorporating fiber-reinforced resin, such as those utilizing long fibers like Ribbond and SFRCs, demonstrated a higher prevalence of desirable fracture patterns [11–13, 32, 35–37]. In the current study, this phenomenon can be attributed to the enhanced stress distribution facilitated by Ribbond fibers placed at the base of the cavity, coupled with the reinforcement of the connection between the buccal enamel wall and the remaining lingual structure, ultimately strengthening the cervical portion of the tooth. The improved performance observed with SFRC can be ascribed to the crack-stopper ability of randomly oriented glass fibers within the composite material. These fibers effectively impede crack propagation toward the core of the restoration and the tooth, thereby alleviating stress concentrations.

There were several limitations in this in vitro study that should be considered when generalizes to clinical situations. This research employed a static force until fracture occurred, whereas in the oral cavity, a combination of static and cyclic masticatory forces can result in the failure of dental restorations and teeth. In addition, the samples of this study were mounted in acrylic resin, and the periodontal ligament that has a crucial role in force distributing in the clinical situations did not reconstruct. Furthermore, the role of temperature fluctuations in the oral environment on water sorption and thermal expansion that could be affected the bonding performance of resin materials to tooth structure should not be neglected. So, more in vitro and clinical studies are needed to clarify the performance of these restoration techniques.

## 5. Conclusion

Based on the results of the present study and considering the mentioned limitations, it can be concluded:

1. The wide and deep MOD cavities in molar teeth can be more strengthened by restoring with SFRC (EverX Posterior) in comparison to resin composite restoration with cusp coverage.
2. The fracture strength and fracture pattern of weaken molars could not be affected by the orientation of long fibers (Ribbond) which were used in the bottom of the resin composite restoration.
3. There was no significant difference between the resin composite restorations with and without cusp coverage.

## Figures and Tables

**Table 1:** The mean and standard deviation (SD) of fracture resistance values in the study groups.

Study groups	Mean ± SD (*N*)*⁣*^*∗*^	Tukey's HSD post hoc test (compared to control group)
G1*⁣*^*∗∗*^	3236.6 ± 370.7^ab^	0.694
G2	4051.4 ± 1293.9^a^	0.845
G3	2886.6 ± 720.4^b^	0.119
G4	3324.5 ± 406.1^ab^	0.849
G5	2949.3 ± 307.5^b^	0.181
G6	3686.7 ± 533.3^ab^	—
One-way ANOVA analysis		*p* = 0.003 *F* = 4.15

*⁣* ^*∗*^The groups that have been marked by different lowercase letters showed significant differences at *p*  < 0.05, based on Tukey HSD test.

*⁣* ^*∗∗*^G1: Conventional resin composite restoration; G2: short fiber-reinforced resin composite restoration; G3 and G4: the Ribbond fibers applied on base of cavity in buccolingual and cross-sectional direction, respectively, followed by conventional resin composite restoration; G5: conventional resin composite restoration with cusp coverage; G6: intact teeth (control).

**Table 2:** The fracture patterns distribution among the study groups (number (percent)).

Fracture pattern	Group					
	G1*⁣*^*∗*^	G2	G3	G4	G5	G6
Favorable/restorable	2 (20%)	6 (60%)	4 (40%)	4 (40%)	0 (0%)	—
Unfavorable/nonrestorable	8 (80%)	4 (40%)	6 (60%)	6 (60%)	10 (100%)	3 (30%)

*⁣* ^*∗*^G1: Conventional resin composite restoration; G2: short fiber-reinforced resin composite restoration; G3 and G4: the Ribbond fibers applied on base of cavity in buccolingual and cross-sectional direction, respectively, followed by conventional resin composite restoration; G5: conventional resin composite restoration with cusp coverage; G6: intact teeth (control).

## Data Availability

The data that support the findings of this study are available on request from the corresponding author. The data are not publicly available due to privacy or ethical restrictions.
